# Letter to the Editor: Long-term dynamic burden and attributable risk analysis of indirect maternal mortality over the past 30 years: an observational study and forecast for perioperative management in the next decade

**DOI:** 10.1097/JS9.0000000000003251

**Published:** 2025-08-21

**Authors:** Yanan Duan, Aiping Chen

**Affiliations:** a Department of Gynecology, The Affiliated Hospital of Qingdao University, Qingdao, Shandong Province, China; b Qingdao Medical College, Qingdao University, Qingdao, Shandong Province, China

*Dear Editor,*

Jiang and colleagues provide a comprehensive panorama of indirect maternal mortality (IMM) across 195 countries, offering valuable insights for perioperative planning in resource-constrained settings[1]. We would like to expand the discussion by addressing the following critical areas that may help refine both policy recommendations and research priorities. The letter to the editor is compliant with the TITAN Guidelines 2025 – governing declaration and use of AI[2].

Firstly, the authors rely on the 2019 Global Burden of Disease (GBD) iteration. However, more recent data from 2021 GBD update and new estimates from the World Health Organization (WHO) extend cause-specific mortality through 2023 and reflect the impact of the COVID-19 pandemic[3]. Between 2019 and 2021, the global maternal mortality ratio increased from 187 to 211 per 100 000 live births, with progress only resumed in 2023[4]. Excluding this period may overstate the apparent decline in IMM in several low- and middle-income regions and may also exaggerate the reported increase in age-standardized death rates in high sociodemographic index settings. Incorporating data from 2020 to 2023 would help clarify whether the observed increase in high income countries reflects real risk or is instead the result of statistical fluctuation around very small numerators.

Secondly, the study commendably identifies the 20–30 age group as bearing the highest IMM burden, this population-level burden may obscure important differences in individual-level risk. In particular, adolescent mothers and those of advanced maternal age often face significantly higher individual risks of complications. These risks arise from physiological and socioeconomic vulnerabilities[5]. It is therefore essential to distinguish between population-level burden and individual-level risk. Future research should employ rigorous age-stratified analyses that meticulously control for parity and socioeconomic factors to accurately identify and target these truly vulnerable subgroups.

Thirdly, the definition of IMM adopted by the authors aggregates all conditions exacerbated by pregnancy, yet the analysis isolates anemia as a primary risk factor. This narrow focus may obscure important interactions among multiple conditions including iron deficiency, malaria, HIV, cardiovascular disease, and surgical complications. Recent comparative-risk modeling suggests that multimorbidity clusters currently account for >40 % of IMM in sub-Saharan Africa and South-East Asia[6]. Disentangling these clusters with joint-attribution methods could better inform integrated antenatal care strategies, rather than targeting micronutrient supplementation alone.

In summary, this important study provides a strong foundation for understanding the global burden of IMM. We suggest that future research could further enhance its impact by incorporating recent data, refining age-related risk assessments, and adopting a more integrated view of contributing health conditions.

## Data Availability

All data are included in this article.

## References

[1] Jiang J Qin L, and Wang B. Long-term dynamic burden and attributable risk analysis of indirect maternal mortality over the past 30 years: an observational study and forecast for perioperative management in the next decade. Int. J. Surg. 10–1097. doi:10.1097/JS9.0000000000002979. PMC12430810 40680008

[2] Agha RA Mathew G Rashid R. Transparency in the reporting of artificial intelligence – the TITAN guideline. Premi J Sci 2025;10:100083.

[3] Seid G Alemu A Diriba G. Routine tuberculosis contact investigation yield and preventive treatment cascade in central Ethiopia. Heliyon 2024;10:e30942. 38770348 10.1016/j.heliyon.2024.e30942 PMC11103515

[4] Organization WH. Trends in Maternal Mortality 2000 to 2023: Estimates by WHO, UNICEF. UNFPA, World Bank Group and UNDESA/Population Division; 2025.

[5] Jeong W Jang S-I Park E-C Nam JY. The effect of socioeconomic status on all-cause maternal mortality: a nationwide population-based cohort study. Int J Environ Res Public Health 2020;17:4606. 32604879 10.3390/ijerph17124606 PMC7345089

[6] Murray CJ Aravkin AY Zheng P. Global burden of 87 risk factors in 204 countries and territories, 1990–2019: a systematic analysis for the Global Burden of Disease Study 2019. Lancet 2020;396:1223–49. 33069327 10.1016/S0140-6736(20)30752-2 PMC7566194

